# Mitochondrial DNA point mutations and relative copy number in 1363 disease and control human brains

**DOI:** 10.1186/s40478-016-0404-6

**Published:** 2017-02-02

**Authors:** Wei Wei, Michael J. Keogh, Ian Wilson, Jonathan Coxhead, Sarah Ryan, Sara Rollinson, Helen Griffin, Marzena Kurzawa-Akinibi, Mauro Santibanez-Koref, Kevin Talbot, Martin R. Turner, Chris-Anne McKenzie, Claire Troakes, Johannes Attems, Colin Smith, Safa Al Sarraj, Christopher M. Morris, Olaf Ansorge, Stuart Pickering-Brown, James W. Ironside, Patrick F Chinnery

**Affiliations:** 1 Institute of Genetic Medicine, Central Parkway, Newcastle University, Newcastle Upon Tyne, NE1 3BZ UK; 2 Institute of Brain, Behaviour and Mental Health, University of Manchester, 2.014 AV Hill Building, Oxford Road, Manchester, M13 9PT UK; 3 Department of Clinical Neurosciences, John Radcliffe Hospital, Level 3, West Wing, Headley Way, Oxford, OX3 9DU UK; 4 National CJD Research & Surveillance Unit, Centre for Clinical Brain Sciences, University of Edinburgh, Western General Hospital, Edinburgh, EH4 2XU UK; 5 Department of Basic and Clinical Neuroscience, Institute of Psychiatry, Psychology and Neuroscience, King’s College London, De Crespigny Park, London, SE5 8AF UK; 6 Department of Neuropathology, John Radcliffe Hospital, West Wing, Level 1, Oxford, OX3 9DU UK; 7 Department of Clinical Neurosciences, University of Cambridge, University Neurology Unit, Level 5 ‘A’ Block, Box 165 Cambridge Biomedical Campus, Cambridge, CB2 0QQ UK; 8 MRC Mitochondrial Biology Unit, Cambridge Biomedical Campus, Cambridge, CB2 0QQ UK

**Keywords:** Mitochondrial, Mutation, Dementia, Neurodegeneration, Somatic

## Abstract

Mitochondria play a key role in common neurodegenerative diseases and contain their own genome: mtDNA. Common inherited polymorphic variants of mtDNA have been associated with several neurodegenerative diseases, and somatic deletions of mtDNA have been found in affected brain regions. However, there are conflicting reports describing the role of rare inherited variants and somatic point mutations in neurodegenerative disorders, and recent evidence also implicates mtDNA levels. To address these issues we studied 1363 *post mortem* human brains with a histopathological diagnosis of Parkinson’s disease (PD), Alzheimer’s disease (AD), Frontotemporal dementia – Amyotrophic Lateral Sclerosis (FTD-ALS), Creutzfeldt Jacob disease (CJD), and healthy controls. We obtained high-depth whole mitochondrial genome sequences using off target reads from whole exome sequencing to determine the association of mtDNA variation with the development and progression of disease, and to better understand the development of mtDNA mutations and copy number in the aging brain. With this approach, we found a surprisingly high frequency of heteroplasmic mtDNA variants in 32.3% of subjects. However, we found no evidence of an association between rare inherited variants of mtDNA or mtDNA heteroplasmy and disease. In contrast, we observed a reduction in the amount of mtDNA copy in both AD and CJD. Based on these findings, single nucleotide variants of mtDNA are unlikely to play a major role in the pathogenesis of these neurodegenerative diseases, but mtDNA levels merit further investigation.

**Electronic supplementary material:**

The online version of this article (doi:10.1186/s40478-016-0404-6) contains supplementary material, which is available to authorized users.

## Introduction

Mitochondria are critical intracellular organelles involved in calcium signaling, lipid biosynthesis, apoptosis [31], and the generation of adenosine triphosphate (ATP) via the mitochondrial respiratory chain [28]. Over the last decade it has become clear that mitochondria play a key role in the pathogenesis of common neurodegenerative disorders. Mitochondria contain their own 16.5 kb circular mitochondria genome (mtDNA), which codes for key components of the mitochondrial proteome. MtDNA is present in 10s-1000s of copies in each cell and undergoes lifelong replication in post-mitotic cells including neurons [10]. With rudimentary mechanisms for repair, mtDNA is vulnerable to mutations, which accumulate within cells and also within the germ line.

There is emerging evidence that genetic variation of mtDNA contributes to the pathogenesis of neurodegenerative disorders [14]. Common population genetic variants divide the human population into a geographically-defined ‘haplogroups’ [30], which have been associated with several neurodegenerative disorders including Alzheimer’s disease (AD) and Parkinson’s disease (PD), conferring a small increase in disease risk [13, 22]. Furthermore, high-density genotyping arrays provide preliminary evidence that rare (minor allele frequency, MAF <5%) mtDNA polymorphisms are also associated with neurodegenerative disease, supporting previous work on AD [12].

In addition to maternally inherited polymorphisms, acquired somatic mutations of mtDNA have also been associated with neurodegenerative disorders. Unlike the maternally inherited germ line variants (which are ‘homoplasmic’), the somatic mutations are usually present alongside the original wild-type molecules (heteroplasmy), and the proportion of mutated alleles determines whether a biochemical defect is manifest at the cellular level. MtDNA deletions accumulate in the ageing brain, reaching higher levels in regions vulnerable to neurodegeneration [3, 26], but evidence describing the accumulation of somatic point mutations or small insertion-deletion mutations (indels) is less compelling, with conflicting reports in the literature [6, 20]. Finally, several recent studies have described abnormal amounts of mtDNA both in cerebrospinal fluid or the brains of patients with neurodegenerative diseases [23, 24], but only in a limited number of individuals.

To provide definitive evidence in all three areas, we studied the entire mtDNA sequence and quantity in1363 *post mortem* brains with Alzheimer’s disease (AD), Amyotrophic-frontotemporal dementia (ALS-FTD), Creutzfeldt-Jakob Disease (CJD), Parkinson’s disease (PD) and Dementia with Lewy Bodies (DLB), and compared them to healthy age-matched control brains. We found no evidence that rare inherited polymorphisms or mtDNA heteroplasmy contributes to the pathogenesis of neurodegenerative diseases, although differences in mtDNA content provide a clue to disease mechanism in AD and CJD.

## Materials and methods

### Study samples

Full mitochondrial genome sequence data was extracted from Exome Sequencing data of 1363 case or control brain tissue samples from the Medical Research Council Brain Tissue Resource [15]. Cases fulfilling both ante-mortem and post-mortem diagnostic criteria for major neurodegenerative disease were included (Table 1, Additional file 1: Methods, Additional file 1: Table S1). DNA was extracted from the cerebellum in 87.3% of cases (*n* = 1190), cerebral cortex in 6.5% of cases (*n* = 89) with other brain regions in 6.16% (*n* = 84).

**Table 1:** Clinical and demographic data of all cases within the study

	Number of cases (n)	Age onset (years)		Age of death (years)		Female		Male	
		Age	SD	Age	SD	n	%	n	%
AD	282	67.0	11.2	78.1	11.6	152	53.9	130	46.1
CJD	181	54.2	19.6	55.1	19.2	96	53.0	85	47.0
Control	351			78.1	12.0	196	55.8	155	44.2
DLB-PD	89	63.9	10.3	75.1	8.5	31	34.8	58	65.2
FTD-ALS	236	58.9	11.2	64.4	11.5	98	41.5	137	58.1
Young Controls	110			41.4	9.7	30	27.3	80	72.7
Other disorders (See Additional file 1: Tables S1-S8)	114	60.3	21.5	72.7	16.7	46	40.3	68	59.7
Mean		60.6	17.2	69.0	17.4		43.9		56.1
Total	1363					598		765	

Key: *AD* Alzheimer’s disease, *CJD* Creutzfeldt Jacob Disease, *DLB-PD* Dementia with Lewy Bodies or Parkinson’s disease, *FTD-ALS* Frontotemporal Dementia or Amyotrophic Lateral Sclerosis. Information about Other disorders can be seen in Additional file 1: Table S2

### Mitochondrial sequences extraction and variant calling

Exome sequencing was performed using Nextera 62 Mb Rapid Capture Expended Exome kits on the Illumina HiSeq 2000 with 100 bp paired-end reads. Sequence data were aligned to the human reference sequence (UCSC hg19) (http://genome.ucsc.edu) using Burrows-Wheeler Aligner (BWA) v0.7.6 [17], formatted using Samtools v0.1.19 [18], and duplicates removed using Picard v1.707 (http://picard.sourceforge.net).

MToolBox [4] recovered mtDNA from off-target sequences, remapped onto the revised Cambridge Reference Sequence [2], and discarded those mapping to regions of (GRCh37/hg19), and considered Nuclear mitochondrial Sequences [29].

Known insertions or deletions (ins/dels) were defined, and all variants scored in HmtDB [27] and MITOMAP [28]. Remaining reads reconstructed the mitochondrial genome. Nucleotide mismatches and ins/del with quality scores (QS ≥25) and read depth (rd ≥5) were included.

### Determining heteroplasmy and homoplasmy

We determined the proportion of variant alleles at each site of the mitochondrial genome. We then calculated the heteroplasmic fraction (HF, %) by dividing the number of variant reads by the total number of reads (for SNVs and deletions) or of the total number of 5′ flanking reads (for insertions). If the HF was <10% or >90%, we conservatively considered the variant site to be homoplasmic. If the HF was between 10 and 90%, the site was considered to be heteroplasmic, and the HF was studied further.

### Defining mtDNA haplogroups

Haplogroup assignment was performed using the mt-classifier tool [32], and a maximum likelihood (ML) phylogenetic tree was created using the PHYLIP software (v3.696) from sequence alignments built by Clustal Omega (v1.2.0) (http://www.clustal.org/omega). Defined haplogroups were compared to the haplogroups genotyped in 2360 individuals from the 1958 Birth Cohort (WTCCC58C) as previously determined by Hudson et al. [11].

### Defining rare variants

Minor allele frequencies for each base of the mitochondrial genome were calculated from 30,506 full-length mitochondrial sequences in NCBI-GenBank using custom Python scripts. Rare homoplasmic variations were defined as those alleles present in less than 5% of individuals within their haplogroup using MITOMASTER [21], and novel variants those not present in the NCBI-GenBank dataset, 1000 genomes [9], MITOMAP [28] or HmDB [27].

### Functional prediction

Pathogenicity scores for all possible non-haplogroup defining SNVs were calculated using MutPred [19] and Polyphen-2 [1].

### MtDNA copy number estimation

The relative amount of mtDNA in each brain (referred to as mtDNA copy number) was calculated as the ratio between the mean mtDNA read depth and the mean exome read depth as previously described [5].

### Statistical analysis

Mean variant counts or fractions both within and between groups were performed using Mann-Whitney, Fisher’s exact, or Spearman’s rank test tests as appropriate and as defined at the uncorrected threshold. A Poisson loglinear model was used to test the association between the number of variants and age among individuals within each main group. All statistical analyses were performed using R (v3.0.2) (http://CRAN.R-project.org/doc/FAQ/R-FAQ.html), and plots made using Python.

## Results

### Coverage and quality of assembled mitochondrial genomes

Complete assembly of the mitochondrial genome was obtained in all cases, with a mean read depth across the genome of 289 (SD = 169.8) (range 66–1328) and a mean base quality score of 36.8 (sd = 0.25) (range 36.0–37.5) (Additional file 1: Figures S1 and S2). There was no difference in mean read depth or mean base quality score for any group *vs* controls (Additional file 1: Figure S3).

### Haplogroup associations

**Fig. 1:**
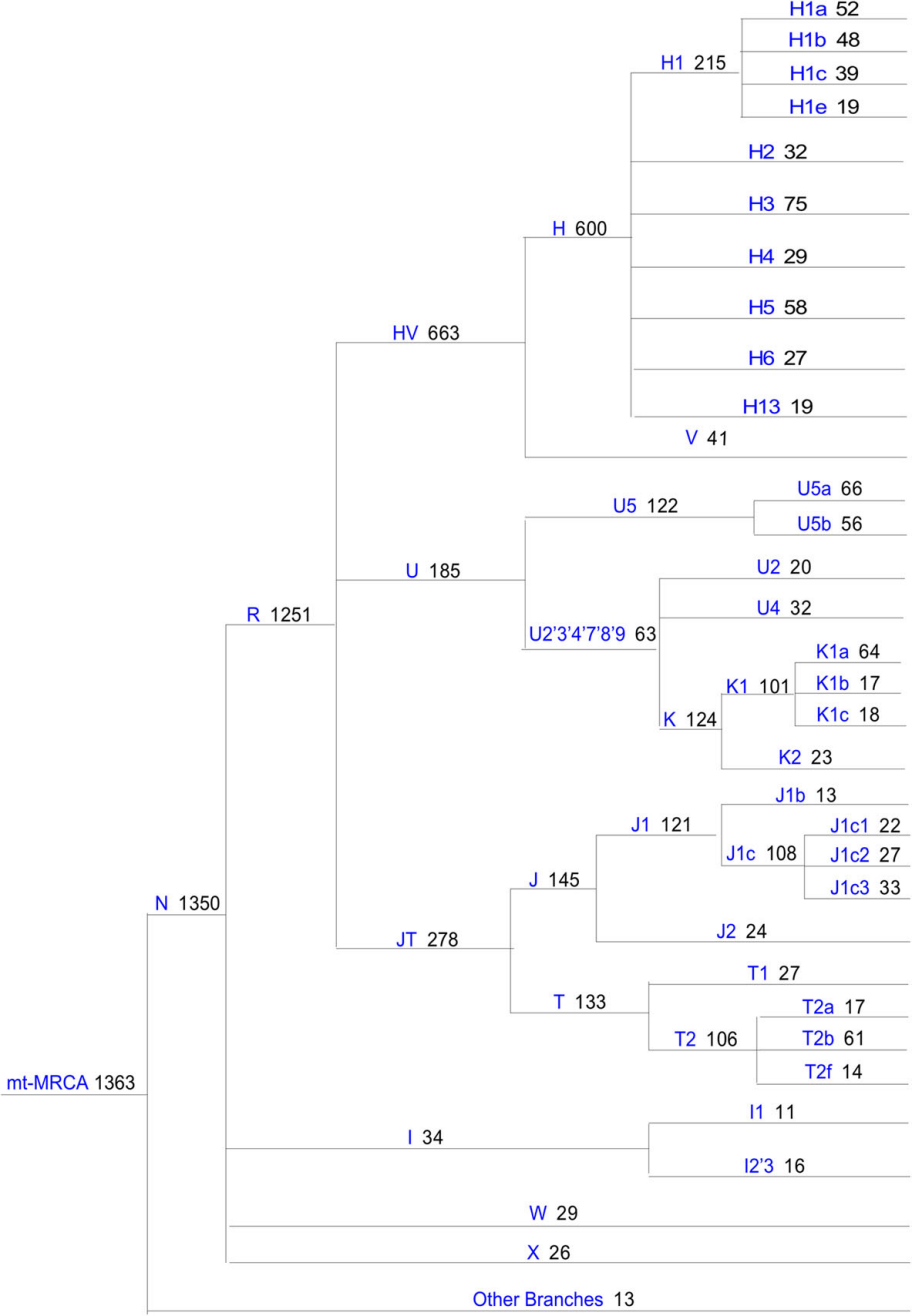
Phylogenetic tree of the 1363 mtDNA sequenced derived from the MRC Brain Tissue Resource. Major haplogroups are shown

Haplogroups and phylogenetic relationships were determined for all 1363 samples (Fig. 1). There was no difference in major overall haplogroup frequency when compared to 2360 UK population controls (Additional file 1: Table S2), confirming the accuracy of haplogroup calling and that the cohort as representative of the UK population. We saw no association between any disease cohort and specific haplogroups in our study (Additional file 1: Table S3).

### Homoplasmic variants

One thousand, nine hundred twenty-three homoplasmic variants were detected within the cohort, with a mean of 22.9 (sd = 10.8) variants per sample. Four hundred sixty-seven variants were defined as ‘common’ (minor population Allele Frequency, MpAF >0.05 within their haplogroup), and included known haplogroup defining variants. One thousand, four hundred fifty-six homoplasmic variants were defined as rare (MpAF <0.05 within their haplogroup). Twenty-five of the rare variants were novel, not seen in the NCBI database (*n* = 30,506), MITOMAP (*n* = 30,589) or the 1000 genomes database. Four of the novel variants were in non-coding regions, two were in rRNA genes, and 19 were synonymous (Additional file 1: Table S4).

### Association with disease – common homoplasmic variants

Here we considered all homoplasmic variants, and a subgroup analysis of all non-synonymous homoplasmic variants. We saw no evidence of a disease association with any single variant, the burden of homoplasmic variants in any gene, nor the burden of homoplasmic variants in groups of genes forming a respiratory chain complex (Additional file 1: Figures S4–S8, Additional file 1: Table S5). However, when stratifying by age, there was a trend towards young onset AD cases (age of death <60) having a greater number of total variants in *MT-TR* compared to controls (6/13 vs 9/139) (*p* = 0.002) (Additional file 1: Figure S5).

### Association with disease - rare homoplasmic variants

No single rare homoplasmic variant was present at greater frequency in any disease compared to controls (Additional file 1: Figure S8). There was a trend towards a greater number of rare homoplasmic point mutations in two genes in AD compared to controls; *MT-RNR1* (AD; 30/282 (10.6%), Controls; 16/344 (4.7%)) (*p* = 0.005)) and again *MT-TR* (AD; 6/282 (2.1%), Controls; 0/344) (*p* = 0.008), although both failed to reach significance at the corrected threshold of *p* = 0.0014. (Additional file 1: Figure S8, Additional file 1: Table S5). When stratified by age, this suggested that the trend towards an excess burden of rare homoplasmic variants in *MT-RNR1* was likely driven by variants in young onset AD cases vs controls (AD: 9/53 (16.9%), Controls (2/65), *p* = 0.012 (3%) (Additional file 1: Figure S9). We also saw that young onset PD-DLB cases (death aged <70) had a significantly greater number of rare homoplastic mutations in *MT-CO2* (PD-DLB: 5/23 (21.7%), Controls: 5/213 (2.3%), *p* = 0.0010 (Additional file 1: Figure S9). The majority of the variants in *MT-CO2* in both cohorts were in non-coding D-loop, but when combined this did not reach the corrected threshold for significance. There was no association between any rare non-synonymous variant, nor the burden of rare non-synonymous variants in any gene or respiratory chain complex in any disease group *vs* controls.

### Heteroplasmic variants

**Fig. 2:**
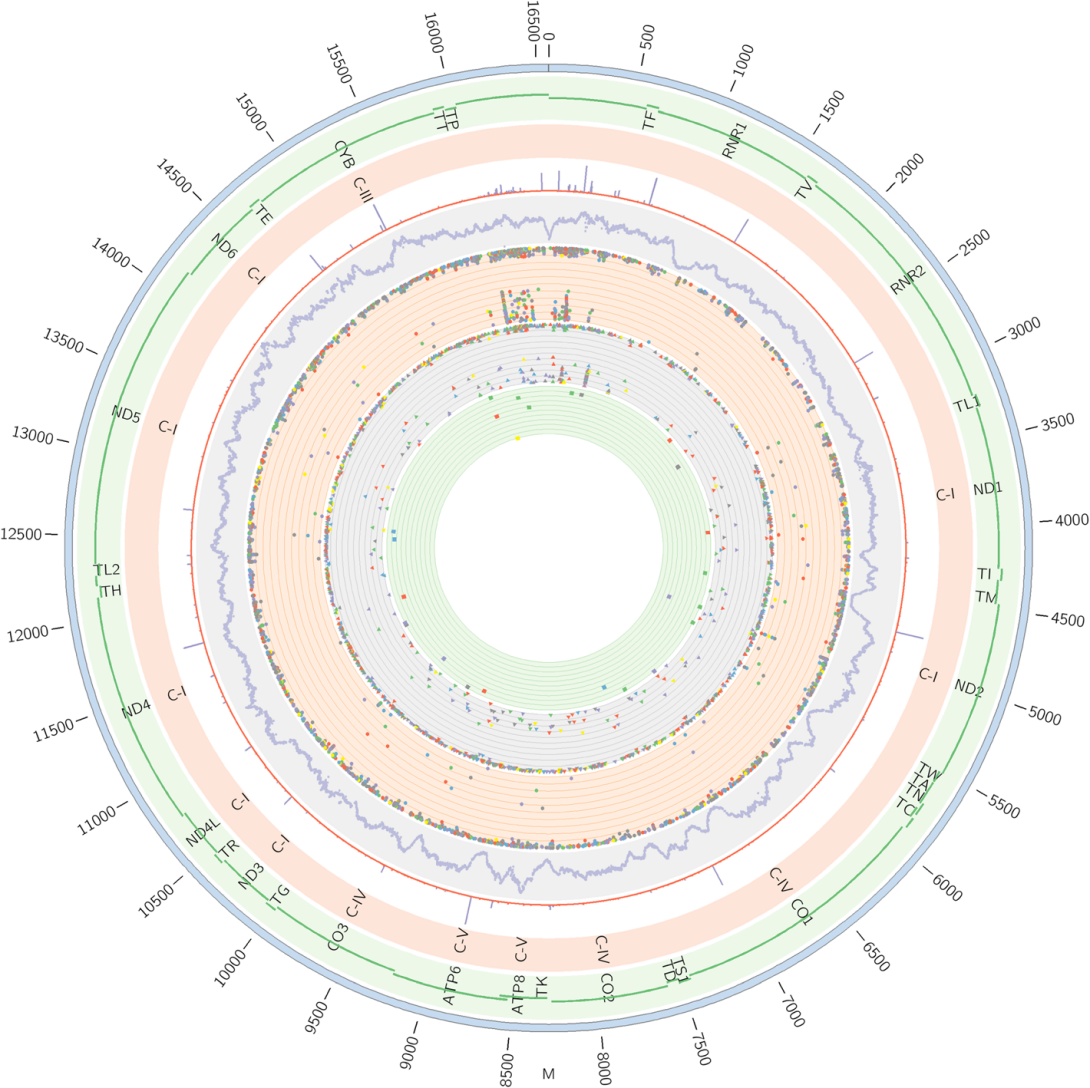
Circos plot summarizing all of the genetic data from 1363 sequences derived form the MRC Brain Tissue Resource. From outside the circle to inside: (1) mtDNA position, (2) mtDNA genes, (3) mtDNA Complex, (4) frequency of variants in 40,440 mitochondrial sequences in NCBI-GenBank, (5) mean read depth of 1363 samples per base, (6) Total variants in all 1363 samples [circles], (7) Total Rare variants in 1363 samples [triangles], (8) Total novel variants in 1363 samples [squares]. Colour code for circles (6) – (8): Red - AD, green - ALS-FTD, blue - CJD, yellow - DLB-PD, grey - others; from inner to outer, HF increasing. Key – AD –Alzheimer’s disease, CJD – Creutzfeldt Jacob Disease, DLB-PD – Dementia with Lewy Bodies or Parkinson’s disease, FTD-ALS – Frontotemporal Dementia or Amyotrophic Lateral Sclerosis

Three hundred eleven heteroplasmic variants (>10% MAF) were detected (mean HF = 7%, sd = 1.0), in 440 cases, with 10 of these variants entirely novel. 55.7% of all heteroplasmic variants occurred within the D-loop, 33.3% in coding regions, 4.8% in rRNA genes and 6.2% in tRNA genes (Fig. 2).

There was no association between any disease group and controls for any single heteroplasmic variant, the total number of heteroplasmic variants, or the mean variant pathogenicity score. There was also no association between the number of non-synonymous heterozygous variants in any gene or complex and any disease group compared to controls (Additional file 1: Tables S6–S8, Additional file 1: Figure S12).

### Heteroplasmy and age

**Fig. 3:**
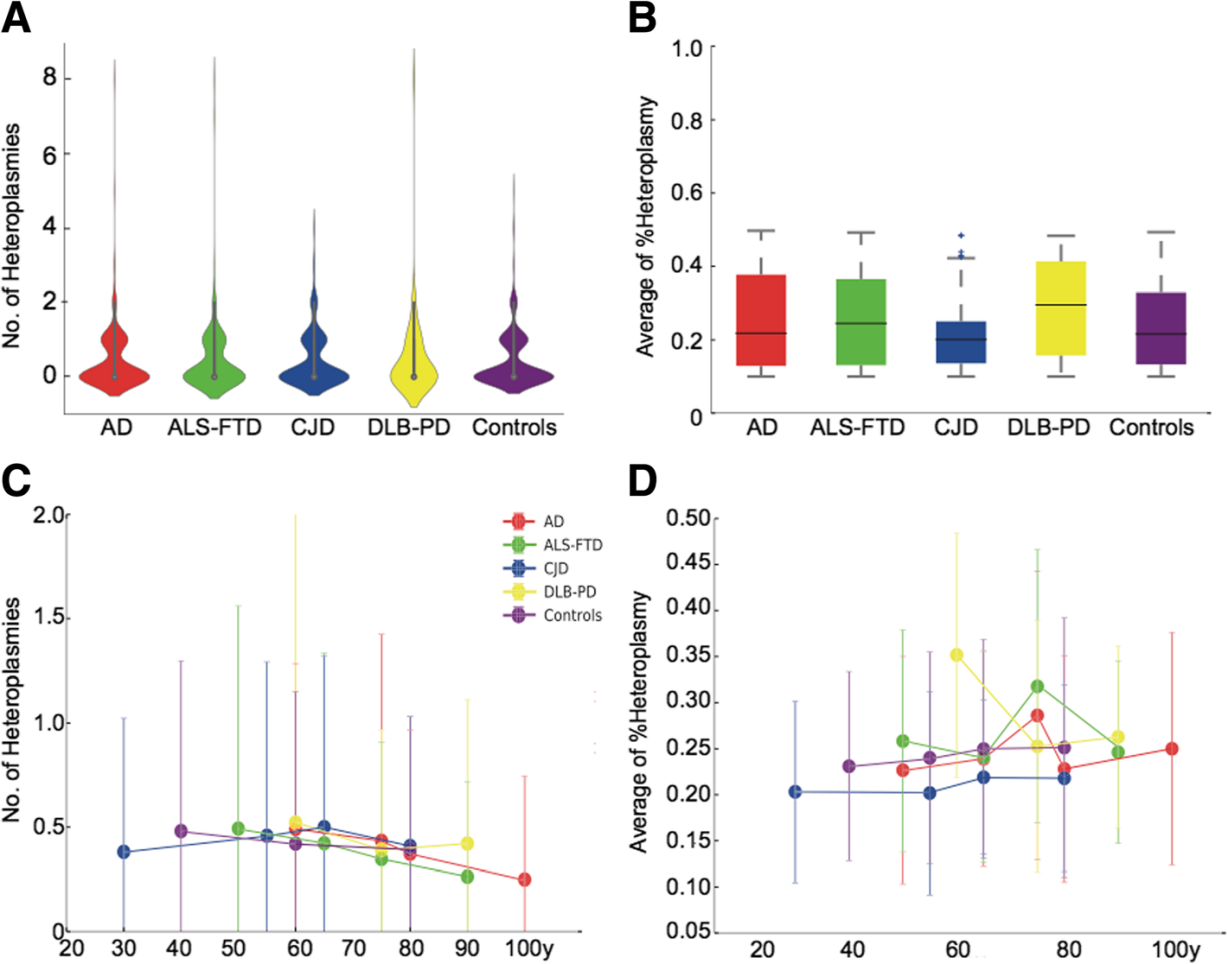
The distribution and nature of major heteroplasmic mtDNA variation within 1363 cases from the MRC Brain Bank Tissue Resource. **a** Top left – The mean number of heteroplasmic variants per sample in each cohort. **b** Top right - The distribution of heteroplasmic fraction (ratio of mutant to wild-type allele) for heteroplasmic variants for each disease cohort. **c** Bottom left – The mean number of heteroplasmic point mutations by age for each disease cohort. **d** Bottom right – The mean heteroplasmic fraction by age for each disease cohort. Key – AD –Alzheimer’s disease, CJD – Creutzfeldt Jacob Disease, DLB-PD – Dementia with Lewy Bodies or Parkinson’s disease, FTD-ALS – Frontotemporal Dementia or Amyotrophic Lateral Sclerosis

We subsequently used a Poison loglinear model to determine the relationship between heteroplasmy and age within each group. There was no age correlation with the total number of heteroplasmic variants, mean level of heteroplasmy (HF), nor the mean variant pathogenicity score in any disease group (Fig. 3).

### mtDNA number

**Fig. 4:**
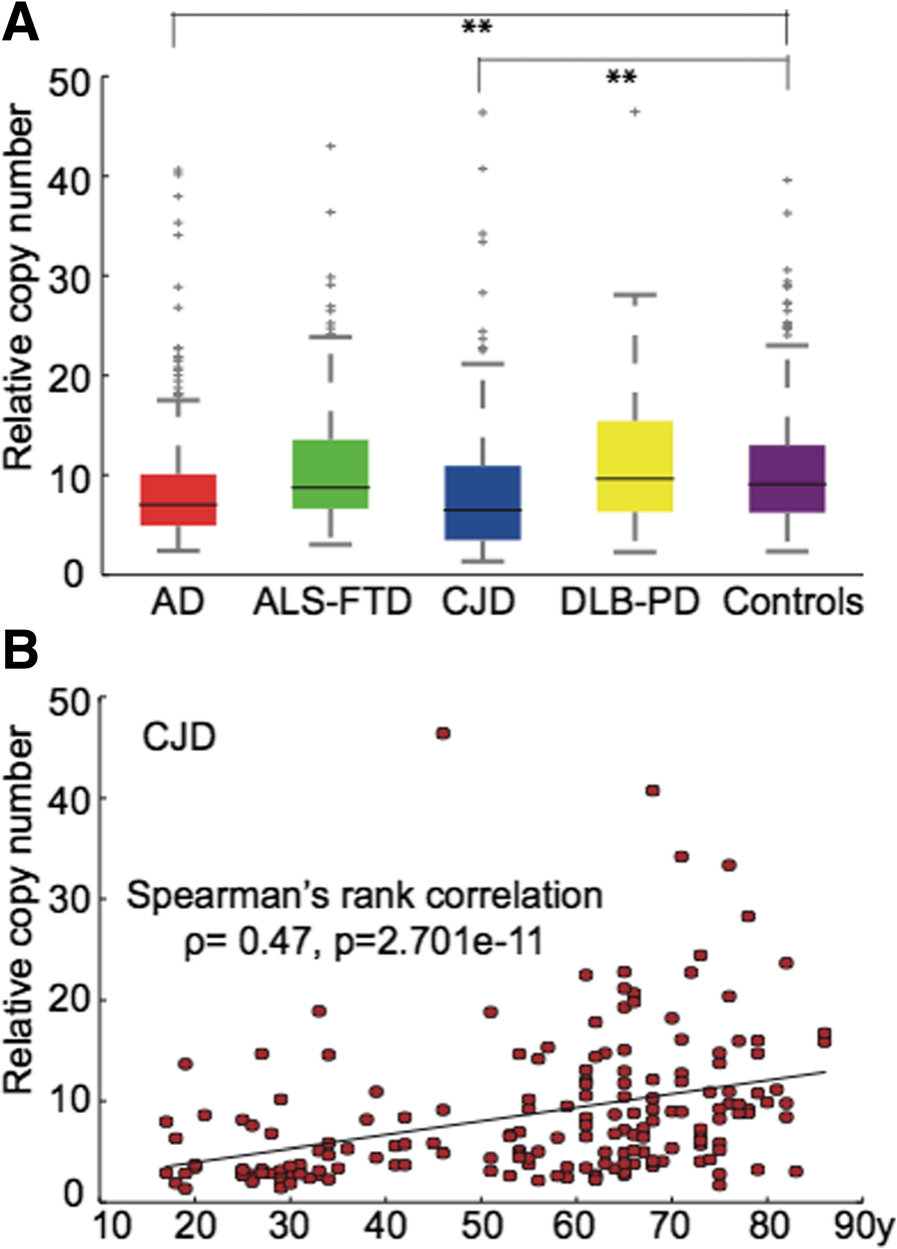
The relative mtDNA copy number in each disease cohort. **a** Top – Relative copy number of each cohort calculated as the ratio between the mean mtDNA read depth and the mean exome read depth as previously described [5]. ** (*P* < 0.01). **b** Bottom - The association between relative mtDNA copy number and age for all CJD cases (*n* = 182) with the Spearman Rank ρ and p-value shown. Key – AD –Alzheimer’s disease, CJD – Creutzfeldt Jacob Disease, DLB-PD – Dementia with Lewy Bodies or Parkinson’s disease, FTD-ALS – Frontotemporal Dementia or Amyotrophic Lateral Sclerosis

mtDNA copy number was significant lower in AD and CJD compared to controls (*p* = 2.85 × 10^−7^ (AD), *p* = 3.34 × 10^−7^ (CJD)), and we observed a strongly positive correlation between age and mtDNA copy number in CJD (*p* = 2.7 × 10^−11^). No association with age was seen in other groups (Fig. 4). The frequency of cerebellar samples in the AD cohort was no different to controls (*p* = 0.64) or the FTD-ALS cohort (*p* = 0.87). However, the CJD cohort did show a greater proportion of cases from the cerebellum compared to all other cohorts (100%, *p* < 0.001 *vs* all other groups).

## Discussion

Here we report a comprehensive study of mtDNA sequence variation and abundance in brain tissue from 1363 neuropathologically characterized *post mortem* samples from MRC Brain Tissue Resource. After correction for multiple significance testing, we saw no difference in the frequency of mtDNA haplogroups, no difference in the frequency of common or rare homoplasmic variants, and no difference in the presence or degree of mtDNA heteroplasmy between different disease groups and control subjects. Overall, we conclude that neither rare homoplasmic variants nor heteroplasmic variants play a substantial role in the pathogenesis of these disorders. However, further work is required to clarify whether mtDNA copy number is important for the pathogenesis of AD and CJD.

Our most interesting finding was the frequent occurrence of mtDNA heteroplasmy in human brain tissue (HF >10% found in 32.3% of cases and controls), but contrary to previous reports, this did not change with age, nor was it associated with disease. This suggests that, although ongoing replication errors in mtDNA occur with age [16, 25], moderate level heteroplasmic variants (>10%) are likely to have either occurred *de novo* in early development, or have been inherited within the germ-line, perhaps clonally expanding to these levels in early life. Resolving this issue will be difficult in human subjects, but will require the analysis of serial samples from the same tissue in the same subject. We cannot exclude the possibility that low level heteroplasmy (MAF <10%) is associated with these different disorders, or that different regions of the brain contain a significant burden of mtDNA variants, as recently described [8]. However, if low level heteroplasmic variants are important (perhaps because the low mean level masks high levels in individual neurons or glia), it is surprising that the higher levels of heteroplasmy we detected were not associated with disease, and that patient brains did not contain more pathogenic mutations than control subjects.

Finally we observed a significant reduction in mtDNA copy number in both Alzheimer’s disease and CJD brains. Our data support previous work in AD [7], but to our knowledge is the first study of mtDNA copy number in CJD brain tissue. It is possible that the CJD finding reflects the higher proportion of cerebellar samples studied when compared to controls. In addition, the mtDNA copy number in the variant CJD (vCJD) cases (*n* = 40, mean mtDNA copy number = 4.14, sd = 2.96) was lower than all other CJD types (mean = 10.03, sd = 7.67; *p* = 2.8 × 10^−6^). Therefore, it is therefore also possible that the trend observed for CJD actually reflects unusually low levels of mtDNA in the brains from patients with vCJD, who died at a younger age than other forms of CJD.

## Conclusion

When taken together, these findings support the role of mtDNA content in the pathogenesis of neurodegeneretive diseases, but with caveats. First, the abnormalities may be restricted to specific brain regions. Previous studies in PD showed a reduction in mtDNA copy number within the *substantia nigra*, but not frontal cortex, raising the possibility that the altered mtDNA levels are a consequence of the underlying pathology, and not the cause. This could be an indirect effect reflecting a change in the proportion of different cell types within a tissue homogenate that is linked to the neurodegeneration. Nonetheless, the observed association between mtDNA copy number and age in CJD brains supports a causal role which requires further investigation. Although this could reflect compensatory mitochondrial biogenesis in older subjects, an alternative explanation is that younger-onset cases have more aggressive pathology, with associated neuronal loss.

## Additional file

Additional file 1: Supplementary Material. (DOCX 6597 kb)
