# Erratum to: Mitochondrial DNA point mutations and relative copy number in 1363 disease and control human brains

**DOI:** 10.1186/s40478-017-0419-7

**Published:** 2017-02-22

**Authors:** Wei Wei, Michael J. Keogh, Ian Wilson, Jonathan Coxhead, Sarah Ryan, Sara Rollinson, Helen Griffin, Marzena Kurzawa-Akanbi, Mauro Santibanez-Koref, Kevin Talbot, Martin R. Turner, Chris-Anne McKenzie, Claire Troakes, Johannes Attems, Colin Smith, Safa Al Sarraj, Christopher M. Morris, Olaf Ansorge, Stuart Pickering-Brown, James W. Ironside, Patrick F Chinnery

**Affiliations:** 1 Institute of Genetic Medicine, Central Parkway, Newcastle University, Newcastle Upon Tyne, NE1 3BZ UK; 2 Institute of Brain, Behaviour and Mental Health, University of Manchester, 2.014 AV Hill Building, Oxford Road, Manchester, M13 9PT UK; 3 Department of Clinical Neurosciences, John Radcliffe Hospital, Level 3, West Wing, Headley Way, Oxford, OX3 9DU UK; 4 National CJD Research & Surveillance Unit, Centre for Clinical Brain Sciences, University of Edinburgh, Western General Hospital, Edinburgh, EH4 2XU UK; 5 Department of Basic and Clinical Neuroscience, Institute of Psychiatry, Psychology and Neuroscience, King’s College London, De Crespigny Park, London, SE5 8AF UK; 6 Department of Neuropathology, John Radcliffe Hospital, West Wing, Level 1, Oxford, OX3 9DU UK; 7 Department of Clinical Neurosciences, University of Cambridge, University Neurology Unit, Level 5 ‘A’ Block, Box 165 Cambridge Biomedical Campus, Cambridge, CB2 0QQ UK; 8 MRC Mitochondrial Biology Unit, Cambridge Biomedical Campus, Cambridge, CB2 0QQ UK

## Erratum

In the original publication of this article [1], the author Marzena Kurzawa-Akinibi was written incorrectly. The correct spelling should have been Marzena Kurzawa-Akanbi. The original article has now been updated to reflect this change.
