# Supplementary material for: Mitochondrial DNA point mutations and relative copy number in 1363 disease and control human brains
Source: Acta Neuropathol Commun. 2017 Feb 2;5:13. doi: 10.1186/s40478-016-0404-6 (PMC5290662; doi:10.1186/s40478-016-0404-6)
Supplement: Supplementary file 1 — Supplementary Material. (DOCX 6597 kb) [file 40478_2016_404_MOESM1_ESM.docx]

**Supplementary Material**

Supplementary Methods - Page 2-4

Supplementary Tables - Page 5-15

Supplementary Figures - Page16 -27

Supplementary References - Page 28

**Supplementary Methods**

**Case cohort allocation**

The *ante mortem* diagnosis was defined by the treating clinician’s working diagnosis in life. The *post mortem* diagnosis was consensus neuropathological diagnosis at death. A clinical vignette of the case, together with a synopsis of the neuropathological report and all quantitative neuropathological criteria (defined below), were also requested and provided when available. All data was reviewed by the study team. Each disease cohort included brains with an *ante mortem* clinical diagnosis consistent with a neurodegenerative disorder and clinical criteria fulfilling the specific neurodegenerative disease at the time of diagnosis. In cases in which a broad phenotype (e.g. ‘dementia’) was recorded, and neuropathology was consistent with a specific diagnosis, they were included within the cohort as defined by their neuropathology. Control cases were defined as those in which a Braak neurofibrillary tangle stage was 2 or lower and there were no other features suggestive of a neurodegenerative disease in either *ante mortem* data, or *post mortem* assessment. Cases ascribed to ‘Vascular disease’ were those in which either there was a history in life suggestive of cognitive impairment with at least one large territorial vascular anomaly such as stroke had occurred, or where Braak stage was unknown. Those ascribed to ‘vascular/control’ showed no large territorial infarcts, no history suggestive of *ante mortem* cognitive impairment and had a Braak stage of less than 2.

All rare neurological diseases, those without *ante mortem* data, or those in which the neuropathology did not fulfill criteria for a specific diagnosis were included into the ‘other’ category and defined by a discrete descriptive term, individual to each case, summarizing the overall clinical and neuropathological phenotype (Supplementary Table 1).

**Neuropathological assessment**

For non-Creutzfelt-Jakob Disease (CJD) cases, additional quantitative neuropathological criteria were obtained when available. These included: Thal phase for Amyloid beta ([Thal et al. 2002](#_ENREF_32)) , Braak neurofibrillary tangle stage ([Braak et al. 2006](#_ENREF_5)), CERAD score ([Mirra et al. 1991](#_ENREF_25)), ABC score ([Montine et al. 2012](#_ENREF_26)), Braak stage for alpha-synuclein ([Braak et al. 2001](#_ENREF_4)), McKeith Lewy body stage ([McKeith et al. 2005](#_ENREF_24)), Cerebral Amyloid Angiopathy (CAA) staging ([Attems et al. 2011](#_ENREF_3)), the presence of TDP-43 pathology and stage ([Mackenzie et al. 2011](#_ENREF_22)), and presence of 3R or 4R tauopathy, fused in sarcoma (FUS) or ubiquitin staining. For cases of CJD, the presence of cerebrospinal fluid (CSF) 14-3-3 protein, and the s100b protein together with MRI imaging features were also requested and provided where available.

**DNA extraction**

Tissue samples (20-30mg) were extracted from Cerebellum (n=1189), Cerebral cortex (lobe undefined) (n=88), Basal ganglia or Caudate (n=7), Muscle (n=1), and undefined brain region (n=78). Automated DNA extraction was performed using a DNA extraction robot (Qiasymphony SP robot; Qiagen, Hilden, Germany). Tissue was lysed in 180 μl of ATL buffer (Qiagen, Hilden, Germany) and 20 μl of Proteinase K (Qiagen, Hilden, Germany). Lysates were incubated overnight at 56 °C and at 900 rpm before being loaded onto the Qiasymphony robot. Subsequent extraction was performed using the Qiasymphony DNA mini kit reagents (Qiagen, Hilden, Germany), as per manufacturers protocol. DNA yield was measured using the Nanodrop-8000 Spectrophotometer (NanoDrop Technologies).

**Exome sequencing – Quality Control**

*Coverage* –To exclude inadvertent duplicate samples, variant calls with a base quality score of 30 and read depth of 10 were converted into PLINK v2.050 ([Purcell et al. 2007](#_ENREF_28)) binary genotyping format using in-house scripts. Pairwise relationships were subsequently determine using KING ([Manichaikul et al. 2010](#_ENREF_23)) allowing for the existence of population structure. Duplicate samples were determined by kinship estimates restricted to first degree or second-degree relatives only using the –related option.

|  | **N** | **Age of onset (years)** | | **Age of death (years)** | | **Female** | **Male** |
| --- | --- | --- | --- | --- | --- | --- | --- |
|  |  | **Mean** | **SD** | **Mean** | **SD** | **N** | **N** |
| **AD and FTD** | 1 |  |  | 73 |  | 0 | 1 |
| **Adult onset gangliosidosis** | 1 | 65 |  | 71 |  | 0 | 1 |
| **Argyrophilic Grain Disease** | 4 | 78 |  | 87.5 | 1.9 | 2 | 2 |
| **Ataxia** | 1 | 85 |  | 85 |  | 1 | 0 |
| **Atypical AD** | 2 | 75 |  | 78.5 | 3.5 | 0 | 2 |
| **Atypical LBD** | 2 | 75 |  | 78.0 | 1.4 | 1 | 1 |
| **Atypical dementia not otherwise classified** | 3 | 55 |  | 77.3 | 11 | 1 | 2 |
| **Atypical Substantia Nigral degeneration** | 2 | 79 |  | 83.5 | 2.1 | 1 | 1 |
| **Atypical tauopathy** | 3 | 75 | 13.1 | 85.3 | 12.5 | 1 | 2 |
| **Atypical TDP-43 deposition** | 1 | 76 |  | 83 |  | 1 | 0 |
| **CADASIL** | 1 | 40 |  | 62 |  | 0 | 1 |
| **Corticobasal Degeneration** | 7 | 62.4 | 11.8 | 72.9 | 12.6 | 1 | 6 |
| **Corticobasal Degeneration and AD** | 1 |  |  | 84 |  | 1 | 0 |
| **Cerebello-olivary atrophy** | 1 |  |  | 75 |  | 1 | 0 |
| **Cerebello-olivary degeneration** | 1 | 12 |  | 58 |  | 1 | 0 |
| **Chorea-acanthocytosis** | 1 |  |  | 40 |  | 0 | 1 |
| **Central Pontine Myelinolysis** | 1 | 64 |  | 67 |  | 0 | 1 |
| **Chronic Traumatic Encephalopathy** | 1 |  |  | 69 |  | 0 | 1 |
| **Demyelination** | 2 |  |  | 43.5 | 13.4 | 1 | 1 |
| **Epilepsy** | 1 |  |  | 24 |  | 1 | 0 |
| **Huntington’s disease** | 6 | 58 | 14.5 | 65.8 | 10.9 | 3 | 3 |
| **Huntington Disease phenocopy** | 1 | 40 |  | 62 |  | 1 | 0 |
| **Hepatic encephalopathy** | 1 |  |  | 52 |  | 1 | 0 |
| **Kuf's disease** | 1 | 43 |  | 58 |  | 0 | 1 |
| **Lewy Body Disease** | 2 |  |  | 90 | 0 | 2 | 0 |
| **Learning difficulty and Epilepsy** | 1 | 10 |  | 54 |  | 0 | 1 |
| **Mild Cognitive Impairment** | 1 |  |  | 85 |  | 0 | 1 |
| **MELAS** | 1 | 13 |  | 49 |  | 0 | 1 |
| **Mitochondrial disease** | 1 | 0 |  | 3 |  | 0 | 1 |
| **Mixed Alzheimer Disease and Lewy Body Disease** | 1 | 63 |  | 75 |  | 1 | 0 |
| **Mixed Corticobasal degeneration and Dementia with Lewy Bodies** | 1 | 79 |  | 77 |  | 0 | 1 |
| **Motor Neuropathy** | 1 |  |  | 90 |  | 0 | 1 |
| **Multiple System Atrophy** | 9 | 58 | 7.4 | 68.6 | 9.5 | 1 | 8 |
| **Multiple Sclerosis** | 3 | 34.3 | 16.7 | 61.7 | 11.9 | 1 | 2 |
| **Neocortical Lewy Body Disease** | 1 |  |  | 73 |  | 0 | 1 |
| **Neuroaxonal dystrophy** | 1 | 14 |  | 16 |  | 1 | 0 |
| **Neurofibrillary tangle only dementia** | 3 | 80.3 | 8 | 87.7 | 9.1 | 0 | 3 |
| **Normal Pressure Hydrocephalus** | 1 | 64 |  | 65 |  | 0 | 1 |
| **Parkinson’s Disease and Motor Neuron Disease** | 1 |  |  |  |  | 0 | 1 |
| **Parkinson’s disease related changes** | 1 |  |  | 87 |  | 0 | 1 |
| **Primary Familial Basal Ganglia Calcification** | 2 | 75 |  | 76 | 2.8 | 2 | 0 |
| **Possible Alzheimer Disease** | 1 | 62 |  | 82 |  | 1 | 0 |
| **Possible paraneoplastic dementia** | 1 | 52 |  | 64 |  | 0 | 1 |
| **Pre-clinical tauopathy** | 1 |  |  | 68 |  | 0 | 1 |
| **Pre-symptomatic Dementia with Lewy Bodies** | 2 | 67 |  | 86.5 | 13.4 | 1 | 1 |
| **Pre-symptomatic Frontotemporal dementia** | 1 |  |  | 79 |  | 1 | 0 |
| **Probable Alzheimer Disease** | 3 | 84 |  | 86.3 | 6.1 | 2 | 2 |
| **Progressive Supranuclear Palsy** | 14 | 68.2 | 12.8 | 78.1 | 10.8 | 8 | 6 |
| **Spinocerebellar ataxia (SCA)** | 1 |  |  | 50 |  | 0 | 1 |
| **SCA1** | 1 |  |  | 76 |  | 0 | 1 |
| **SCA14** | 1 |  |  | 103 |  | 0 | 1 |
| **SCA2** | 2 |  |  | 68 | 18.4 | 1 | 1 |
| **SCA7** | 1 |  |  | 58 |  | 1 | 0 |
| **Spinal muscular atrophy** | 1 |  |  |  |  | 1 | 0 |
| **Tauopathy** | 4 | 63 | 1.4 | 72.8 | 11.2 | 2 | 3 |
| **Uncategorized dementia** | 1 |  |  | 95 |  | 1 | 0 |
| **Unusual tauopathy** | 1 |  |  | 67 |  | 0 | 1 |
| **Vascular disease / DLB** | 1 | 90 |  | 91 |  | 1 | 0 |
|  |  |  |  |  |  |  |  |

**Supplementary Table 1.** All cases classified within the ‘Other disorders’ category within Table 1. All demographic data, gender, and age of onset and death are recorded, together with the diagnosis as judged by the clinic-pathological consensus at post-mortem.

| **Major haplogroup** | **WTCCC - 58C** | **Our study** | **P-value** |
| --- | --- | --- | --- |
|  |  |  |  |
| **H** | 1160 (43.9) | 600 (44.0) | 0.97 |
| **V** | 84 (3.2) | 41 (3.0) | 0.85 |
| **J** | 311 (11.8) | 145 (10.6) | 0.29 |
| **T** | 266 (10.1) | 133 (9.8) | 0.78 |
| **U** | 334 (12.7) | 185 (13.6) | 0.43 |
| **K** | 241 (9.1) | 124 (9.1) | 1.00 |
| **W** | 46 (1.7) | 29 (2.1) | 0.39 |
| **X** | 46 (1.7) | 26 (1.9) | 0.71 |
| **I** | 99 (3.8) | 34 (2.5) | 0.041 |

**Supplementary Table 2.** Comparison between the haplogroup frequencies of the Wellcome Trust Case Control Consortium 1958 Birth Cohort (WTCCC-58C) (n=2360 cases) as determined by Hudson et al [1], and the haplogroup frequency in our study (n=1363). All associations were performed using a Fisher’s exact test, and are shown at the uncorrected threshold. We observed a slightly lower frequency of haplogroup I individuals in our study compared to the WTCCC-58C, though otherwise all the other major haplogroups showed similar frequencies between cohorts.

|  | **Old Controls** | **AD** |  | **ALS-FTD** |  | **CJD** |  | **DLB-PD** |  | **Young Controls** | |
| --- | --- | --- | --- | --- | --- | --- | --- | --- | --- | --- | --- |
| **Main Haplogroup** | **n=234** | **n=282** | **p-value** | **n=236** | **p-value** | **n=181** | **p-value** | **n=89** | **p-value** | **n=110** | **p-value** |
| **H** | 105 | 118 | 0.53 | 119 | 0.27 | 78 | 0.77 | 48 | 0.17 | 55 | 0.42 |
| **I** | 10 | 6 | 0.20 | 4 | 0.11 | 4 | 0.29 | 1 | 0.30 | 4 | 1.00 |
| **J** | 30 | 33 | 0.79 | 26 | 0.57 | 19 | 0.54 | 6 | 0.17 | 8 | 0.14 |
| **K** | 16 | 25 | 0.42 | 16 | 1.00 | 17 | 0.36 | 9 | 0.35 | 7 | 1.00 |
| **T** | 26 | 35 | 0.68 | 16 | 0.11 | 23 | 0.65 | 8 | 0.69 | 8 | 0.33 |
| **U** | 28 | 40 | 0.51 | 31 | 0.78 | 22 | 1.00 | 11 | 1.00 | 18 | 0.31 |
| **V** | 6 | 11 | 0.46 | 8 | 0.79 | 5 | 1.00 | 0 | 0.19 | 7 | 0.13 |
| **W** | 6 | 3 | 0.31 | 6 | 1.00 | 6 | 0.77 | 2 | 1.00 | 1 | 0.44 |
| **X** | 2 | 6 | 0.30 | 6 | 0.28 | 2 | 1.00 | 3 | 0.13 | 2 | 0.60 |
| **Others(A,C,D,L,M,N&R)** | 5 | 5 | 1.00 | 4 | 1.00 | 5 | 0.51 | 1 | 1.00 | 0 | 0.31 |

**Supplementary Table 3.** Haplogroup breakdown and association compared to old control cohort (n=234) for each of the major disease cohorts. Key: AD – Alzheimer’s disease, ALS-FTD – Amyotophic Lateral Sclerosis – Frontotemporal Dementia, CJD – Creutzfeldt Jacob Disease, DLB-PD – Dementia with Lewy Bodies – Parkinson’s disease

| **No.** | **Phenotype** | **Age of disease onset** | **Age of death** | **Variant** | **Amino acid change** |
| --- | --- | --- | --- | --- | --- |
| 1. | AD | Unknown | 78 | m.2221C>T | rRNA |
| 2. | AD | Unknown | 88 | m.3885C>T | syn:T=>T |
| 3. | AD | Unknown | 90 | m.9404A>G | syn:T=>T |
| 4. | AD | Unknown | 92 | m.11578C>T | syn:S=>S |
| 5. | AD | 58 | 67 | m.15577C>T | syn:A=>A |
| 6. | ALS-FTD | 67 | 80 | m.383T>C | non-coding |
| 7. | ALS-FTD | Unknown | 37 | m.4556C>T | syn:T=>T |
| 8. | ALS-FTD | Unknown | 54 | m.5117C>T | syn:F=>F |
| 9. | ALS-FTD | Unknown | 71 | m.5399C>T | syn:N=>N |
| 10. | ALS-FTD | Unknown | 73 | m.6986A>C | syn:S=>S |
| 11. | ALS-FTD | Unknown | 70 | m.15598C>T | syn:V=>V |
| 12. | ALS-FTD | 17 | 18 | m.16040C>T | non-coding |
| 13. | ALS-FTD | 36 | 43 | m.16198T>C | non-coding |
| 14. | CJD | 55 | 58 | m.7287C>T | syn:L=>L |
| 15. | CJD | 26 | 27 | m.12576C>T | syn:F=>F |
| 16. | CJD | 61 | 61 | m.12709C>T | syn:L=>L |
| 17. | DLB-PD | 67 | 72 | m.15838C>T | syn:I=>I |
| 18. | PSP | Unknown | 77 | m.550A>C | non-coding |
| 19. | DLB-PD | Unknown | 73 | m.2658T>C | rRNA |
| 20. | Atypical substantia nigral degeneration | Unknown | 85 | m.9683A>G | syn:M=>M |
| 21. | Vascular Disease | 58 | 85 | m.11131C>T | syn:T=>T |
| 22. | Controls | Unknown | 57 | m.6419A>G | syn:K=>K |
| 23. | Controls | Unknown | 56 | m.10293C>T | syn:L=>L |
| 24. | Controls | Unknown | 48 | m.5150C>T | syn:T=>T |
| 25 | Controls | Unknown | 46 | m.11161C>T | syn:T=>T |

**Supplementary Table 4.** Clinical and pathological details of all patients with novel homoplasmic variants. All cases had both clinical and pathological confirmation of their respective diagnoses. Key: AD – Alzheimer’s disease, ALS-FTD – Amyotophic Lateral Sclerosis – Frontotemporal Dementia, CJD – Creutzfeldt Jacob Disease, DLB-PD – Dementia with Lewy Bodies – Parkinson’s disease, PSP – Progressive Supranuclear Palsy. Syn – synonymous. Amino-acid changes are coded by single-letter amino-acid code.

|  | **Controls (n=344)** | **AD (n=282)** | | **ALS-FTD (n=236)** | | **CJD (n=181)** | | **DLB-PD (n=89)** | |
| --- | --- | --- | --- | --- | --- | --- | --- | --- | --- |
| **Loci** | **n** | **n** | **p-value** | **n** | **p-value** | **n** | **p-value** | **n** | **p-value** |
| **Complex1** | 163 | 140 | 0.575 | 109 | 0.800 | 85 | 1.000 | 50 | 0.154 |
| **Complex3** | 49 | 53 | 0.129 | 41 | 0.350 | 21 | 0.421 | 14 | 0.737 |
| **Complex4** | 83 | 74 | 0.579 | 50 | 0.423 | 43 | 1.000 | 28 | 0.174 |
| **Complex5** | 53 | 40 | 0.735 | 34 | 0.813 | 26 | 0.798 | 16 | 0.626 |
| **MT-ATP6** | 45 | 35 | 0.811 | 29 | 0.801 | 19 | 0.483 | 11 | 1.000 |
| **MT-ATP8** | 8 | 5 | 0.781 | 6 | 1.000 | 7 | 0.408 | 6 | 0.047* |
| **MT-CO1** | 41 | 36 | 0.807 | 28 | 1.000 | 24 | 0.677 | 11 | 0.857 |
| **MT-CO2** | 17 | 22 | 0.183 | 10 | 0.842 | 11 | 0.683 | 11 | 0.026* |
| **MT-CO3** | 37 | 26 | 0.594 | 17 | 0.190 | 13 | 0.213 | 9 | 1.000 |
| **MT-CYB** | 49 | 53 | 0.129 | 41 | 0.350 | 21 | 0.421 | 14 | 0.737 |
| **MT-DLOOP** | 115 | 109 | 0.181 | 92 | 0.186 | 76 | 0.057 | 41 | 0.035* |
| **MT-NC1** | 1 | 0 | 1.000 | 2 | 0.570 | 0 | 1.000 | 0 | 1.000 |
| **MT-NC3** | 2 | 0 | 0.504 | 1 | 1.000 | 0 | 0.547 | 0 | 1.000 |
| **MT-NC5** | 1 | 1 | 1.000 | 0 | 1.000 | 1 | 1.000 | 0 | 1.000 |
| **MT-NC7** | 2 | 0 | 0.504 | 0 | 0.517 | 0 | 0.547 | 0 | 1.000 |
| **MT-ND1** | 30 | 34 | 0.186 | 29 | 0.165 | 13 | 0.617 | 9 | 0.679 |
| **MT-ND2** | 50 | 43 | 0.822 | 34 | 1.000 | 25 | 0.896 | 5 | 0.031* |
| **MT-ND3** | 6 | 12 | 0.090 | 7 | 0.396 | 5 | 0.524 | 3 | 0.399 |
| **MT-ND4** | 40 | 42 | 0.236 | 28 | 1.000 | 17 | 0.465 | 11 | 0.854 |
| **MT-ND4L** | 11 | 7 | 0.639 | 7 | 1.000 | 10 | 0.241 | 2 | 1.000 |
| **MT-ND5** | 62 | 51 | 1.000 | 33 | 0.211 | 33 | 1.000 | 21 | 0.231 |
| **MT-ND6** | 30 | 15 | 0.120 | 16 | 0.437 | 9 | 0.160 | 13 | 0.112 |
| **MT-RNR1** | 16 | 30 | 0.005* | 15 | 0.453 | 4 | 0.231 | 2 | 0.549 |
| **MT-RNR2** | 28 | 17 | 0.352 | 19 | 1.000 | 12 | 0.606 | 7 | 1.000 |
| **MT-TA** | 0 | 0 | 1.000 | 1 | 0.407 | 0 | 1.000 | 0 | 1.000 |
| **MT-TC** | 2 | 1 | 1.000 | 1 | 1.000 | 0 | 0.547 | 0 | 1.000 |
| **MT-TD** | 1 | 1 | 1.000 | 0 | 1.000 | 0 | 1.000 | 0 | 1.000 |
| **MT-TE** | 2 | 0 | 0.504 | 2 | 1.000 | 0 | 0.547 | 0 | 1.000 |
| **MT-TF** | 3 | 3 | 1.000 | 2 | 1.000 | 1 | 1.000 | 0 | 1.000 |
| **MT-TG** | 2 | 0 | 0.504 | 1 | 1.000 | 1 | 1.000 | 0 | 1.000 |
| **MT-TH** | 2 | 3 | 0.662 | 2 | 1.000 | 3 | 0.346 | 0 | 1.000 |
| **MT-TI** | 2 | 3 | 0.662 | 1 | 1.000 | 0 | 0.547 | 0 | 1.000 |
| **MT-TK** | 2 | 0 | 0.504 | 0 | 0.517 | 1 | 1.000 | 0 | 1.000 |
| **MT-TL1** | 0 | 0 | 1.000 | 0 | 1.000 | 1 | 0.345 | 0 | 1.000 |
| **MT-TL2** | 2 | 1 | 1.000 | 0 | 0.517 | 0 | 0.547 | 0 | 1.000 |
| **MT-TM** | 2 | 1 | 1.000 | 3 | 0.401 | 0 | 0.547 | 0 | 1.000 |
| **MT-TN** | 0 | 0 | 1.000 | 0 | 1.000 | 2 | 0.118 | 0 | 1.000 |
| **MT-TP** | 0 | 0 | 1.000 | 2 | 0.165 | 0 | 1.000 | 0 | 1.000 |
| **MT-TQ** | 1 | 0 | 1.000 | 0 | 1.000 | 1 | 1.000 | 0 | 1.000 |
| **MT-TR** | 0 | 6 | 0.008* | 2 | 0.165 | 4 | 0.014* | 0 | 1.000 |
| **MT-TS1** | 1 | 0 | 1.000 | 0 | 1.000 | 0 | 1.000 | 0 | 1.000 |
| **MT-TS2** | 0 | 0 | 1.000 | 0 | 1.000 | 2 | 0.118 | 0 | 1.000 |
| **MT-TT** | 6 | 7 | 0.581 | 5 | 0.764 | 2 | 0.721 | 2 | 0.670 |
| **MT-TV** | 1 | 0 | 1.000 | 0 | 1.000 | 0 | 1.000 | 0 | 1.000 |
| **MT-TW** | 1 | 3 | 0.332 | 2 | 0.570 | 2 | 0.274 | 0 | 1.000 |
| **MT-TY** | 0 | 3 | 0.091 | 0 | 1.000 | 0 | 1.000 | 0 | 1.000 |

**Supplementary Table 5.** The number of patients with each disorder with rare homoplasmic variants within each complex or gene. P<0.05* (uncorrected threshold). No associations were significant at the corrected threshold (Complexes: p=0.0125, Individual genes: p=0.00135). Gene names are given in the first column. MT-NC1, MT-NC3, MT-NC5, MT-NC7 are non-coding nucleotides.Key - AD – Alzheimer’s disease, ALS-FTD – Amyotophic Lateral Sclerosis – Frontotemporal Dementia, CJD – Creutzfeldt Jacob Disease, DLB-PD – Dementia with Lewy Bodies – Parkinson’s disease.

|  | **Control (n=344)** | | **AD (n=282)** | | | | **ALS-FTD (n=236)** | | | | **CJD (n=181)** | | | | **DLB-PD (n=89)** | | | |
| --- | --- | --- | --- | --- | --- | --- | --- | --- | --- | --- | --- | --- | --- | --- | --- | --- | --- | --- |
|  | **All** | **Rare** | **All** |  | **Rare** |  | **All** |  | **Rare** |  | **All** |  | **Rare** |  | **All** |  | **Rare** |  |
| **Loci** | **n** | **n** | **n** | **p-value** | **n** | **p-value** | **n** | **p-value** | **n** | **p-value** | **n** | **p-value** | **n** | **p-value** | **n** | **p-value** | **n** | **p-value** |
| **Complex1** | 23 | 13 | 18 | 1.000 | 12 | 0.839 | 18 | 0.742 | 14 | 0.235 | 16 | 0.385 | 8 | 0.815 | 7 | 0.645 | 3 | 1.000 |
| **Complex3** | 6 | 5 | 3 | 0.524 | 2 | 0.467 | 4 | 1.000 | 3 | 1.000 | 1 | 0.431 | 1 | 0.669 | 1 | 1.000 | 1 | 1.000 |
| **Complex4** | 13 | 10 | 11 | 1.000 | 10 | 0.656 | 8 | 1.000 | 7 | 1.000 | 8 | 0.815 | 4 | 0.780 | 3 | 1.000 | 3 | 0.735 |
| **Complex5** | 4 | 2 | 1 | 0.385 | 1 | 1.000 | 5 | 0.497 | 4 | 0.230 | 1 | 0.664 | 1 | 1.000 | 2 | 0.608 | 2 | 0.189 |
| **MT-ATP6** | 3 | 2 | 1 | 0.631 | 1 | 1.000 | 3 | 0.691 | 3 | 0.401 | 0 | 0.555 | 0 | 0.547 | 1 | 1.000 | 1 | 0.499 |
| **MT-ATP8** | 1 | 0 | 0 | 1.000 | 0 | 1.000 | 2 | 0.570 | 1 | 0.407 | 1 | 1.000 | 1 | 0.345 | 1 | 0.369 | 1 | 0.206 |
| **MT-CO1** | 7 | 5 | 4 | 0.762 | 4 | 1.000 | 3 | 0.747 | 3 | 1.000 | 3 | 1.000 | 1 | 0.669 | 2 | 1.000 | 2 | 0.636 |
| **MT-CO2** | 3 | 2 | 4 | 0.707 | 4 | 0.417 | 1 | 0.649 | 1 | 1.000 | 1 | 1.000 | 1 | 1.000 | 2 | 0.274 | 2 | 0.189 |
| **MT-CO3** | 3 | 3 | 3 | 1.000 | 2 | 1.000 | 4 | 0.451 | 3 | 0.691 | 4 | 0.241 | 2 | 1.000 | 0 | 1.000 | 0 | 1.000 |
| **MT-CYB** | 6 | 5 | 3 | 0.524 | 2 | 0.467 | 4 | 1.000 | 3 | 1.000 | 1 | 0.431 | 1 | 0.669 | 1 | 1.000 | 1 | 1.000 |
| **MT-DLOOP** | 70 | 24 | 58 | 1.000 | 16 | 0.623 | 38 | 0.232 | 6 | 0.021* | 38 | 0.910 | 14 | 0.727 | 15 | 0.550 | 6 | 1.000 |
| **MT-NC3** | 0 | 0 | 0 | 1.000 | 0 | 1.000 | 0 | 1.000 | 0 | 1.000 | 1 | 0.345 | 0 | 1.000 | 0 | 1.000 | 0 | 1.000 |
| **MT-NC7** | 0 | 0 | 1 | 0.450 | 1 | 0.450 | 0 | 1.000 | 0 | 1.000 | 0 | 1.000 | 0 | 1.000 | 0 | 1.000 | 0 | 1.000 |
| **MT-ND1** | 3 | 2 | 4 | 0.707 | 2 | 1.000 | 4 | 0.451 | 3 | 0.401 | 2 | 1.000 | 1 | 1.000 | 1 | 1.000 | 0 | 1.000 |
| **MT-ND2** | 6 | 3 | 3 | 0.524 | 2 | 1.000 | 3 | 0.745 | 2 | 1.000 | 4 | 0.743 | 1 | 1.000 | 2 | 0.670 | 0 | 1.000 |
| **MT-ND3** | 1 | 1 | 2 | 0.591 | 0 | 1.000 | 3 | 0.309 | 2 | 0.570 | 3 | 0.121 | 1 | 1.000 | 1 | 0.369 | 1 | 0.369 |
| **MT-ND4** | 3 | 3 | 3 | 1.000 | 3 | 1.000 | 1 | 0.649 | 1 | 0.649 | 2 | 1.000 | 2 | 1.000 | 0 | 1.000 | 0 | 1.000 |
| **MT-ND4L** | 1 | 0 | 0 | 1.000 | 0 | 1.000 | 1 | 1.000 | 1 | 0.407 | 0 | 1.000 | 0 | 1.000 | 0 | 1.000 | 0 | 1.000 |
| **MT-ND5** | 7 | 3 | 4 | 0.762 | 3 | 1.000 | 5 | 1.000 | 4 | 0.451 | 4 | 1.000 | 2 | 1.000 | 4 | 0.248 | 2 | 0.274 |
| **MT-ND6** | 3 | 1 | 3 | 1.000 | 2 | 0.591 | 3 | 0.691 | 2 | 0.570 | 1 | 1.000 | 1 | 1.000 | 1 | 1.000 | 0 | 1.000 |
| **MT-RNR1** | 1 | 1 | 0 | 1.000 | 0 | 1.000 | 3 | 0.309 | 3 | 0.309 | 2 | 0.274 | 1 | 1.000 | 2 | 0.109 | 2 | 0.109 |
| **MT-RNR2** | 1 | 0 | 5 | 0.096 | 4 | 0.041* | 2 | 0.570 | 1 | 0.407 | 2 | 0.274 | 2 | 0.118 | 1 | 0.369 | 1 | 0.206 |
| **MT-TA** | 0 | 0 | 0 | 1.000 | 0 | 1.000 | 1 | 0.407 | 0 | 1.000 | 0 | 1.000 | 0 | 1.000 | 0 | 1.000 | 0 | 1.000 |
| **MT-TC** | 1 | 1 | 0 | 1.000 | 0 | 1.000 | 0 | 1.000 | 0 | 1.000 | 0 | 1.000 | 0 | 1.000 | 0 | 1.000 | 0 | 1.000 |
| **MT-TF** | 0 | 0 | 0 | 1.000 | 0 | 1.000 | 0 | 1.000 | 0 | 1.000 | 0 | 1.000 | 0 | 1.000 | 1 | 0.206 | 1 | 0.206 |
| **MT-TI** | 1 | 0 | 0 | 1.000 | 0 | 1.000 | 0 | 1.000 | 0 | 1.000 | 0 | 1.000 | 0 | 1.000 | 0 | 1.000 | 0 | 1.000 |
| **MT-TL1** | 0 | 0 | 1 | 0.450 | 1 | 0.450 | 0 | 1.000 | 0 | 1.000 | 0 | 1.000 | 0 | 1.000 | 0 | 1.000 | 0 | 1.000 |
| **MT-TL2** | 0 | 0 | 0 | 1.000 | 0 | 1.000 | 1 | 0.407 | 1 | 0.407 | 0 | 1.000 | 0 | 1.000 | 0 | 1.000 | 0 | 1.000 |
| **MT-TM** | 0 | 0 | 1 | 0.450 | 0 | 1.000 | 0 | 1.000 | 0 | 1.000 | 0 | 1.000 | 0 | 1.000 | 0 | 1.000 | 0 | 1.000 |
| **MT-TQ** | 1 | 1 | 0 | 1.000 | 0 | 1.000 | 0 | 1.000 | 0 | 1.000 | 0 | 1.000 | 0 | 1.000 | 0 | 1.000 | 0 | 1.000 |
| **MT-TR** | 3 | 0 | 1 | 0.631 | 0 | 1.000 | 5 | 0.281 | 1 | 0.407 | 5 | 0.131 | 0 | 1.000 | 0 | 1.000 | 0 | 1.000 |
| **MT-TS1** | 1 | 1 | 0 | 1.000 | 0 | 1.000 | 0 | 1.000 | 0 | 1.000 | 0 | 1.000 | 0 | 1.000 | 0 | 1.000 | 0 | 1.000 |
| **MT-TT** | 1 | 0 | 1 | 1.000 | 0 | 1.000 | 1 | 1.000 | 0 | 1.000 | 1 | 1.000 | 1 | 0.345 | 1 | 0.369 | 0 | 1.000 |
| **MT-TW** | 0 | 0 | 0 | 1.000 | 0 | 1.000 | 0 | 1.000 | 0 | 1.000 | 1 | 0.345 | 1 | 0.345 | 0 | 1.000 | 0 | 1.000 |

**Supplementary Table 6.** The number of total (all) and rare (MAF < 5% in their haplogroup reference databases) heteroplasmic variants. The number of variants in each complex and gene are shown within each disease cohort and the p-value for a Fisher’s exact test vs Controls (n=351) calculated and shown. Key – VAF – Variant Allele Frequency, AD – Alzheimer’s disease, ALS-FTD – Amyotophic Lateral Sclerosis – Frontotemporal Dementia, CJD – Creutzfeldt Jacob Disease, DLB-PD – Dementia with Lewy Bodies – Parkinson’s disease. Mt-NC3, MT-NC7 are non-coding nucleotides.

|  | **Controls** | **AD** |  | **ALS-FTD** |  | **CJD** |  | **DLB-PD** |  |
| --- | --- | --- | --- | --- | --- | --- | --- | --- | --- |
| **Loci** |  | **n=282** | **p-value** | **n=236** | **p-value** | **n=181** | **p-value** | **n=89** | **p-value** |
| **MT-ATP6** | 2 | 0 | 0.504 | 2 | 1.000 | 0 | 0.547 | 0 | 1.000 |
| **MT-ATP8** | 0 | 0 | 1.000 | 1 | 0.407 | 1 | 0.345 | 1 | 0.206 |
| **MT-CO1** | 3 | 3 | 1.000 | 3 | 0.691 | 1 | 1.000 | 0 | 1.000 |
| **MT-CO2** | 2 | 4 | 0.417 | 1 | 1.000 | 0 | 0.547 | 1 | 0.499 |
| **MT-CO3** | 1 | 1 | 1.000 | 3 | 0.309 | 4 | 0.050 | 0 | 1.000 |
| **MT-CYB** | 6 | 1 | 0.136 | 2 | 0.483 | 1 | 0.431 | 1 | 1.000 |
| **MT-ND1** | 0 | 4 | 0.041 | 4 | 0.027 | 1 | 0.345 | 0 | 1.000 |
| **MT-ND2** | 1 | 1 | 1.000 | 0 | 1.000 | 0 | 1.000 | 0 | 1.000 |
| **MT-ND3** | 0 | 2 | 0.203 | 0 | 1.000 | 1 | 0.345 | 0 | 1.000 |
| **MT-ND4** | 0 | 1 | 0.450 | 1 | 0.407 | 2 | 0.118 | 0 | 1.000 |
| **MT-ND4L** | 1 | 0 | 1.000 | 0 | 1.000 | 0 | 1.000 | 0 | 1.000 |
| **MT-ND5** | 1 | 1 | 1.000 | 4 | 0.164 | 1 | 1.000 | 2 | 0.109 |
| **MT-ND6** | 2 | 2 | 1.000 | 1 | 1.000 | 1 | 1.000 | 0 | 1.000 |
| **Complex1** | 5 | 11 | 0.073 | 10 | 0.059 | 6 | 0.200 | 2 | 0.636 |
| **Complex3** | 6 | 1 | 0.136 | 2 | 0.483 | 1 | 0.431 | 1 | 1.000 |
| **Complex4** | 6 | 8 | 0.421 | 7 | 0.396 | 5 | 0.524 | 1 | 1.000 |
| **Complex5** | 2 | 0 | 0.504 | 3 | 0.401 | 1 | 1.000 | 1 | 0.499 |

**Supplementary Table 7.** All non-synonymous heteroplasmic variants. The number of non-synonymous heteroplasmic variants in each complex and gene are shown within each disease cohort and the p-value for a Fisher’s exact test vs Controls (n=351) calculated and shown. Key – VAF – Variant Allele Frequency, AD – Alzheimer’s disease, ALS-FTD – Amyotophic Lateral Sclerosis – Frontotemporal Dementia, CJD – Creutzfeldt Jacob Disease, DLB-PD – Dementia with Lewy Bodies – Parkinson’s disease.

|  | **Controls** | **AD** |  | **ALS-FTD** |  | **CJD** |  | **DLB-PD** |  |
| --- | --- | --- | --- | --- | --- | --- | --- | --- | --- |
| **Loci** |  | **n=282** | **p-value** | **n=236** | **p-value** | **n=181** | **p-value** | **n=89** | **p-value** |
| **MT-ATP6** | 2 | 0 | 0.504 | 2 | 1.000 | 0 | 0.547 | 0 | 1.000 |
| **MT-ATP8** | 0 | 0 | 1.000 | 1 | 0.407 | 1 | 0.345 | 1 | 0.206 |
| **MT-CO1** | 2 | 3 | 0.662 | 3 | 0.401 | 1 | 1.000 | 0 | 1.000 |
| **MT-CO2** | 1 | 4 | 0.180 | 1 | 1.000 | 0 | 1.000 | 1 | 0.369 |
| **MT-CO3** | 1 | 1 | 1.000 | 2 | 0.570 | 2 | 0.274 | 0 | 1.000 |
| **MT-CYB** | 5 | 0 | 0.068 | 1 | 0.409 | 1 | 0.669 | 1 | 1.000 |
| **MT-ND1** | 0 | 2 | 0.203 | 3 | 0.067 | 0 | 1.000 | 0 | 1.000 |
| **MT-ND2** | 1 | 1 | 1.000 | 0 | 1.000 | 0 | 1.000 | 0 | 1.000 |
| **MT-ND3** | 0 | 0 | 1.000 | 0 | 1.000 | 1 | 0.345 | 0 | 1.000 |
| **MT-ND4** | 0 | 1 | 0.450 | 1 | 0.407 | 2 | 0.118 | 0 | 1.000 |
| **MT-ND4L** | 0 | 0 | 1.000 | 0 | 1.000 | 0 | 1.000 | 0 | 1.000 |
| **MT-ND5** | 1 | 1 | 1.000 | 3 | 0.309 | 1 | 1.000 | 2 | 0.109 |
| **MT-ND6** | 1 | 2 | 0.591 | 1 | 1.000 | 1 | 1.000 | 0 | 1.000 |
| **Complex1** | 3 | 7 | 0.123 | 8 | 0.058 | 5 | 0.131 | 2 | 0.274 |
| **Complex3** | 5 | 0 | 0.068 | 1 | 0.409 | 1 | 0.669 | 1 | 1.000 |
| **Complex4** | 4 | 8 | 0.151 | 6 | 0.330 | 3 | 0.697 | 1 | 1.000 |
| **Complex5** | 2 | 0 | 0.504 | 3 | 0.401 | 1 | 1.000 | 1 | 0.499 |

**Supplementary Table 8.** All rare non-synonymous heteroplasmic variants (MAF <5% within their haplogroup). The number of rare non-synonymous heteroplasmic variants in each complex and gene are shown within each disease cohort and the p-value for a Fisher’s exact test vs Controls (n=351) calculated and shown. Key – VAF – Variant Allele Frequency, AD – Alzheimer’s disease, ALS-FTD – Amyotophic Lateral Sclerosis – Frontotemporal Dementia, CJD – Creutzfeldt Jacob Disease, DLB-PD – Dementia with Lewy Bodies – Parkinson’s disease.

**Supplementary Figures**


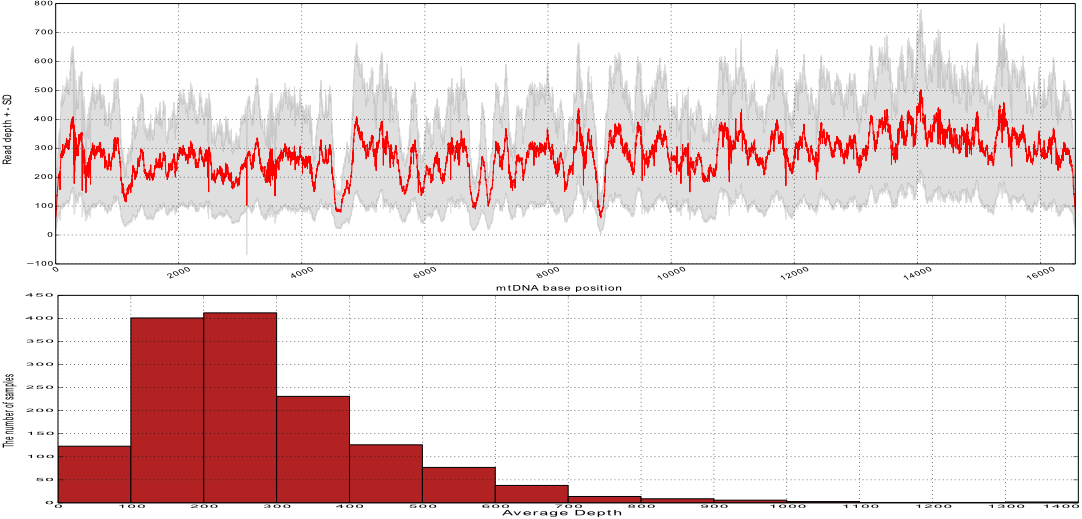


**Supplementary Figure 1** Distribution of read depth across the mitochondrial genome. Top panel – mean read depth from all 1363 cases by base (red line) with standard deviation of red depth (grey area). Bottom panel – A histogram representing the number of samples and their mean coverage across the whole mitochondrial genome.


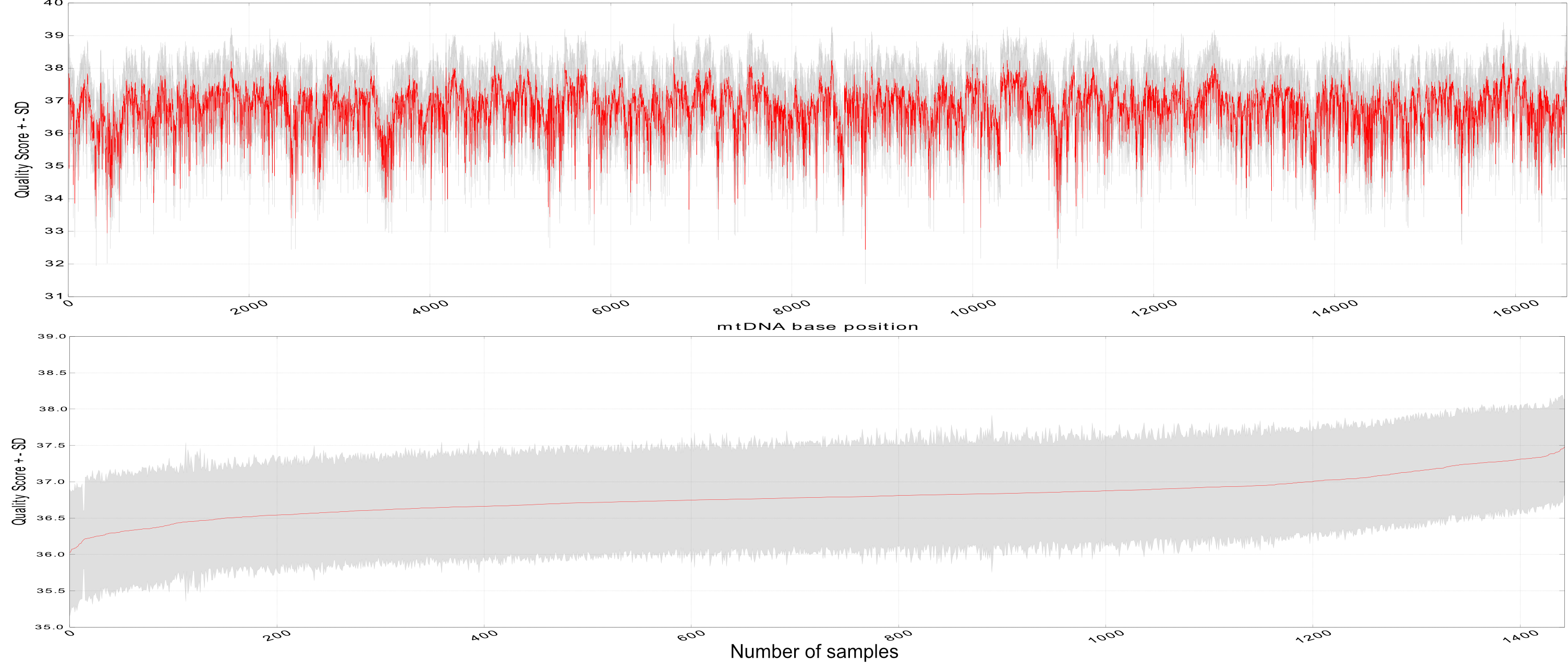


**Supplementary Figure 2.** Distribution of base quality scores across the mitochondrial genome. Top panel – the mean per base quality score (red line), with standard deviation (grey area). Bottom panel – the mean quality score against the number of samples within the cohort.

**Supplementary Figure 3.** Box-whisker plots of the distribution of whole mitochondrial genome read depth (A) and quality score (B) by cohort. No differences were observed between any disease group and controls for either parameter.

**Supplementary Figure 4.** All homoplasmic variants in the major disease cohorts compared to controls. A – The association between cases and controls for each homoplasmic variant is shown, with each disease cohort represented by colour. Uncorrected p-value thresholds are shown. B - Case vs control analysis of all homoplasmic variants, clustered by gene. The –log 10 p value for each cohort against controls is shown. The corrected p-value threshold is p=0.00135, or –log10 p value of 2.85.

**Supplementary Figure 5.** Case vs control analysis of all homoplasmic variants, clustered by gene, and stratified by age. The –log 10 p value for each cohort against controls is shown. Each disease cohort is depicted by a specific colour, and age range by symbol shape (left panel). The corrected p-value threshold is p=0.00135, or –log10 p value of 2.85

**Supplementary Figure 6.** All non-synonymous homoplasmic variants in the major disease cohorts compared to controls. A – The association between cases and controls for each non-synonymous homoplasmic variant is shown, with each disease cohort represented by colour. Uncorrected p-value thresholds are shown. B – A box-whisker plot of the distribution of non-synonymous variants in each cohort. There were no significant differences between groups (data not shown).

**Supplementary Figure 7.** All non-synonymous homoplasmic variants by gene (A) and by gene and stratified by age (B). A - A case vs control analysis of all non-synonymous homoplasmic variants, clustered by gene. The –log 10 p value for each cohort against controls is shown. Each disease cohort is depicted by a specific colour again. The corrected p-value threshold is (p=0.0038, or –log10 p value of 2.42) B - The –log 10 p value for each cohort against controls is shown. Each disease cohort is depicted by a specific colour, and age range by symbol shape (left panel). The corrected p-value threshold is (p=0.0038, or –log10 p value of 2.42).

**Supplementary Figure 8**. All rare homoplasmic variants in the major disease cohorts compared to controls. A – The association between cases and controls for each rare homoplasmic variant is shown, with each disease cohort represented by colour. Uncorrected p-value thresholds are shown. B Case vs control analysis of all rare homoplasmic variants, clustered by gene. The –log 10 p value for each cohort against controls is shown. Each disease cohort is depicted by a specific colour. The corrected p-value threshold is p=0.00135, or –log10 p value of 2.85.

**Supplementary Figure 9.** All rare homoplasmic variants in the major disease cohorts stratified by age. Case vs control analysis of all rare homoplasmic variants, clustered by gene. The –log 10 p value for each cohort against controls is shown. Each disease cohort is depicted by a specific colour. The corrected p-value threshold for genes is p=0.00135, or –log10 p value of 2.85

**Supplementary Figure 10**. All rare non-synonymous homoplasmic variants. A case control analysis of all rare non-synonymous homoplasmic variants for cases vs controls . A – The association between cases and controls for each rare non-synonymous homoplasmic variant, with each disease cohort represented by colour. Uncorrected p-value thresholds are shown. B - Case vs control analysis of all rare non-synonymous homoplasmic variants clustered by gene. The –log 10 p value for each cohort against controls is shown. Each disease cohort is depicted by a specific colour. The corrected p-value threshold for genes is p=0.0038, or –log10 p value of 2.42

**Supplementary Figure 11.** All rare non-synonymous homoplasmic variants in the major disease cohorts stratified by age. Case vs control analysis of all rare non-synonymous homoplasmic variants, clustered by gene. The –log 10 p value for each cohort against controls is shown. Each disease cohort is depicted by a specific colour. The corrected p-value threshold is p=0.0038, or –log10 p value of 2.42.

**Supplementary Figure 12.** Distribution and nature of heteroplasmic variants in the major disease cohorts. A – All single heteroplasmic SNPs in each disease cohorts vs controls. Each disease cohort is coded by colour, with –log10 p-values for each cohort vs controls shown. B – All heteroplasmic variants clustered by gene and stratified by age vs controls. Again -log10 p-values are shown for each disease group (coded by colour) and age cohort (coded by symbol). The corrected p-value threshold is p=0.00135, or –log10 p value of 2.85.

**Supplementary References**

1 Gleeson JG, Minnerath S, Kuzniecky RIet al. (2000) Somatic and germline mosaic mutations in the doublecortin gene are associated with variable phenotypes. American journal of human genetics 67: 574-581 Doi 10.1086/303043
